# Saline-induced changes of epicuticular waxy layer on the *Puccinellia tenuiflora* and *Oryza sativa* leave surfaces

**DOI:** 10.1186/s40659-015-0023-x

**Published:** 2015-07-01

**Authors:** Chunxue Yang, Shurong Ma, Imshik Lee, Jaineung Kim, Shenkui Liu

**Affiliations:** Key Laboratory of Saline-alkali Vegetation Ecology Restoration in Oil Field (SAVER), Ministry of Education, Alkali Soil Natural Environmental Science Center (ASNESC), Northeast Forestry University, Harbin, 150040 China; Institute of Physics, Nankai University, Tianjin, 130071 China; Deparment of Packaging Engineering, Yonsei University, Wonju, Kangwon-do 220-710 South Korea

**Keywords:** Saline tolerance, Morphology, Wax crystal, Element analysis, Na^+^ localization

## Abstract

**Background:**

The epicuticular waxy layer of plant leaves enhances the extreme environmental stress tolerance. However, the relationship between waxy layer and saline tolerance was not established well. The epicuticular waxy layer of rice (*Oryza sativa L.*) was studied under the NaHCO_3_ stresses. In addition, strong saline tolerance *Puccinellia tenuiflora* was chosen for comparative studies.

**Results:**

Scanning electron microscope (SEM) images showed that there were significant changes in waxy morphologies of the rice epicuticular surfaces, while no remarkable changes in those of *P. tenuiflora* epicuticular surfaces. The NaHCO_3_-induced morphological changes of the rice epicuticular surfaces appeared as enlarged silica cells, swollen corns-shapes and leaked salt columns under high stress. Energy dispersive X-ray (EDX) spectroscopic profiles supported that the changes were caused by significant increment and localization of [Na^+^] and [Cl^−^] in the shoot. Atomic absorption spectra showed that [Na^+^]_shoot_/[Na^+^]_root_ for *P. tenuiflora* maintained stable as the saline stress increased, but that for rice increased significantly.

**Conclusion:**

In rice, NaHCO_3_ stress induced localization and accumulation of [Na^+^] and [Cl^−^] appeared as the enlarged silica cells (MSC), the swollen corns (S-C), and the leaked columns (C), while no significant changes in *P. tenuiflora*.

## Background

Soil salination has become an important factor that restricts agricultural development across the globe. Saline soil takes up 37 % of the world’s arable land [1]. Saline regions in China are mostly composed of Na_2_CO_3_ and NaHCO_3_. Up-to-date researchers have focused salt tolerance study on NaCl, but rare on alkali salt. The threats posed by alkali salt are much more complex and destructive to the ecosystem than by neutral salt [2].

The waxy layer that covers over plant surfaces plays an important role in natural package, which serves as the first barrier to protect plants against threats from the external environment [2, 3]. The waxy layer helps protect plants against non-biological stress such as non-stomatal water loss, insect intrusion [4], bacterial invasion, ultraviolet radiation and frost [5]. This natural wax mechanism brings new insight not only for environmental and agricultural applications, but also for the industrial application in biomimetics-package. The waxy surface varies from plant to plant [6–8]. Content of plant waxy is determined not only genetically, but also influenced by the environment. The environmental factors have an impact on the biochemical process of waxy synthesis. Correlation between epidermal waxy deposits and drought tolerance has been found in various plants [8, 9]. However, there was rare research on the relationship between plants’ waxy layer and their saline tolerance [10].

*P. tenuiflora* is a perennial grass of the Gramineae family and has extremely strong saline tolerance, which is used as a pioneer plant in the improvement of saline soils. The waxy surfaces of the *p. tenuiflora* leaves have always been a controversial issue with regard to a salt-secreting structure [10]. The excess salt in *P. tenuiflora* could be discharged through the formation of the waxy layer. However, it is unclear how exactly do the changes of the waxy layer respond to different degrees of saline stress.

Rice (*O. sativa L*.) is one of the most widely consumed foods as well as the second-highest production of food over the world. Rice has a medium saline tolerance. The epicuticular surface of rice shoots is composed of epidermis cell, stomatal guard cell, trichome, and wart-like protuberance (silicon cell) with crystalline wax layers. There is a salt tolerance wild rice but no cultivated rice that has the discharge of the excessive salts [11].

In this work, we selected *P. tenuiflora* and rice, for the further studies in dynamic characteristics of the epicuticular waxy formations in terms of different exposures of NaHCO_3_ stress. The changes in the waxy ornamentation of epicuticular surface of *P. tenuiflora* and rice leaves under NaHCO_3_ stress were visualized by using environmental scanning electron microscope (ESEM) and their chemical composition were analyzed by X-ray diffraction (XRD) analysis. The relationship between epicuticular waxy layer and saline tolerance was explored based on the observations.

## Results

### Morphologies of the epicuticular surfaces

**Fig. 1:**
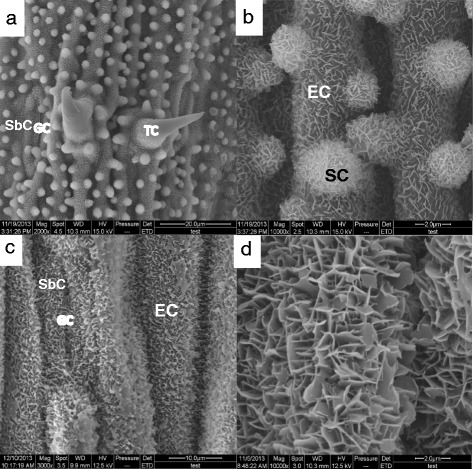
ESEM visualization of the epicuticular rice surfaces under the normal conditions. **a** Rice leaf epicuticular surface containing epidermis (EC), guard cell (GC), silica cell (SC), subsidiary cell (SbC), and trichome (TC). Bar = 20 μm. **b** Magnified ESEM image of rice leaf to visualize the crystalline wax platelet networks on the epicuticular surfaces. Bar = 2 μm. **c** *P. tenuiflora* leaf epidermis. Bar = 10 μm. **d** Magnified ESEM image of *P. tenuiflora* leaf surface. Bar = 2 μm

Figure 1 showed the typical ESEM images of the epicuticular surfaces of rice and *P. tenuiflora* leaves. The epicuticular surface of rice leaves (Fig. 1a and b) contained epidermis cell (EC), stomatal guard cell (GC), stomatal subsidiary cell (SbC), trichome (TC), and wart-like protuberance (silica cell, SC). Crystalline wax covered over the epicuticular surfaces. Wax crystals appeared as randomly distributed crystals over the epicuticular surfaces (Fig. 1b). The wax crystals showed no specific orientation, and their planes were standing with acute angles to the epicuticular surface. The random orientation of small-sized crystalline waxes formed the micro-networks. The heights of platelet wax crystals were less than 0.2 μm. There was no noticeable difference of crystalline wax layers on EC, GC, SbC, and SCs, but no on TC.

Figure 1c and d showed the similar wax crystals on the epicuticular surface of the *P. tenuiflora* leaves. It seemed that there were two different sized crystals in the waxy networks (Fig. 1d). Smaller crystals formed more dense networks within the networks formed by bigger crystals. There was no distinction of waxy morphology between on EC, GC, and SbC. There were no SC and TC on the *P. tenuiflora* leaves. The density of the wax crystal networks in *P. tenuiflora* was higher than that in rice.

### NaHCO_3_ stress induced changes of rice epicuticular surfaces

**Fig. 2:**
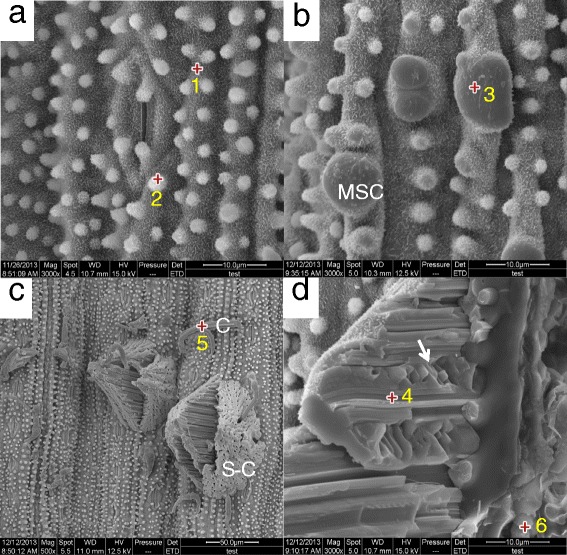
ESEM photographs of rice epicuticular surfaces; (**a**) under no NaHCO_3_ stress. Bar = 10 μm. **b** Under 100 mM NaHCO_3_ for 5 days exposure. Bar = 10 μm. Merged/enlarged silica cells (MSCs) were newly formed. **c** Under 100 mM NaHCO_3_ stress for 7 days exposure. Bar = 50 μm. Swollen corn-shapes (S-Cs) and columns (Cs) appeared. **d** Magnified ESEM image of cross-section of a swollen corn-shape Under 200 mM NaHCO_3_ stress for 5 days exposure. Bar = 10 μm. S-C. Cubic crystals appeared as marked arrow. The marked numbers are the spots for EDX microanalysis

As NaHCO_3_ stress increased for the rice samples, the epicuticular morphologies changed (Fig. 2). Figure 2b showed that wart-like protuberance silica cells merged and enlarged to be the big protuberances (MSC). The distribution density of wax crystal networks deceased and disappeared on the apex surfaces of the MSC. At 100 mM NaHCO_3_ with 7 days exposure, leaked columns (C) and/or swollen corn-shapes (S-Cs) appeared on the surface (Fig. 2c). Interestingly, wax crystals remained on the surfaces of the S-Cs. Diameter of the leaked columns was 2 ~ 5 μm, while the size of the swollen corn was bigger than 10 μm. Cracked side view of the S-Cs revealed the cubic crystals as marked arrow in Fig. 2d, indicating NaCl crystals. There were also the solidified particles underneath of the cell wall as marked number 6 on Fig. 2d.

### EDX element analysis of the epicuticular surface of rice leaves

**Fig. 3:**
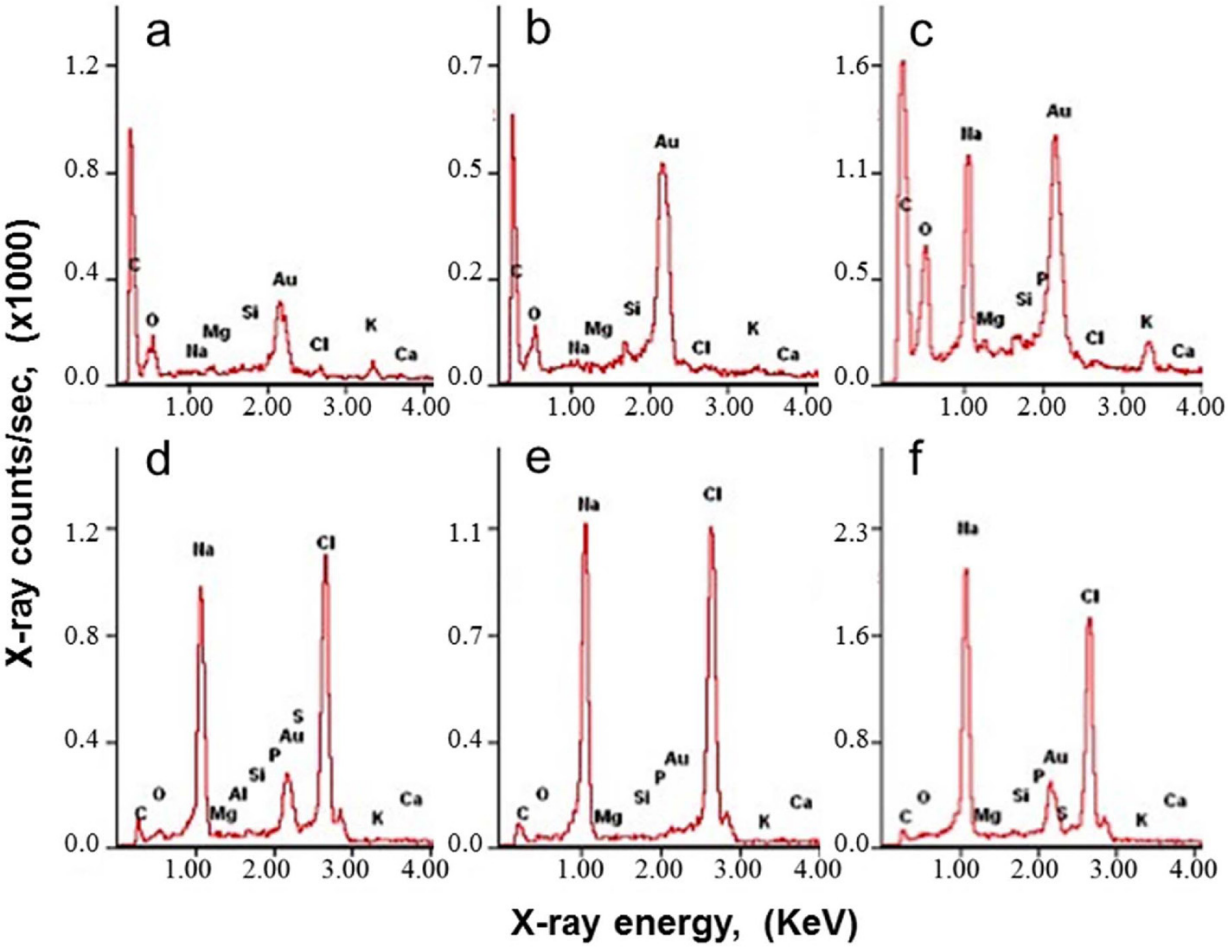
EDX microanalysis spectra of rice leaves. The spectra obtained from; (**a**) EC under no NaHCO_3_ stress (#1 spot in Fig. 2a), (**b**) SC under no NaHCO_3_ stress (#2 spot in Fig. 2a), (**c**) MSC under 100 mM NaHCO_3_ for 5 days exposure (#3 spot in Fig. 2b), (**d**) S-C under 200 mM NaHCO_3_ for 5 days exposure (#4 spot in Fig. 2d), (**e**) C under 100 mM NaHCO_3_ for 7 days exposure (#5 spot in Fig. 2c), and (**f**) The solidified particles underneath of the cell wall under 200 mM NaHCO_3_ for 5 days exposure (#6 spot in Fig. 2d)

EDX microanalysis spectroscopies were obtained from the different spots over the epicuticular surfaces as marked numbers in Fig. 2. For the controlled rice leave surfaces, there were no significant difference among the EDX spectroscopies obtained from SbC, EC and SC. C and O elements (Fig. 3a and b) were dominated. Traces of other elements including gold were also detected. Relatively high gold peak came from the gold coating. The level of silicon accumulation was low on both epidermal region and silica cells. For the NaHCO_3_ exposed rice, there were significant changes in Na and Cl counts at the points on the merged and enlarged silica cells (MSC) (Fig. 3c), the swollen corn shapes (S-C) (Fig. 3d) and the leaked columns (C) (Fig. 3e). Weight %s of Na and Cl on MSC were counted 12.5 % and 0.3 %, respectively. EDX spectra from the localized swollen corn surfaces showed that concentrations of Na and Cl were 20 ~ 30 time higher than those form the normal controlled surfaces. The particles underneath cell wall also showed high counts of Na and Cl (Fig. 3f). The cubic crystals on cross-section surface of the swollen corn appeared, indicating NaCl crystal. At the higher saline stress, condensed Na and Cl were leaked trough the ruptured surfaces to form the NaCl columns. There was more excessive Cl^−^than Na^+^ on the swollen corns, while excessive Na^+^ than Cl^−^ on the leaked columns.

**Fig. 4:**
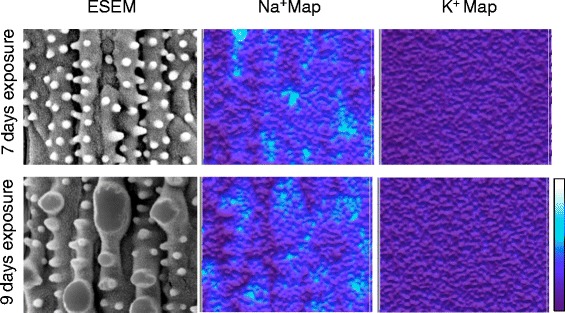
EDX element contour map over rice leaf surfaces for 7 days and 9 days exposure of 100 mM NaHCO_3_. Na localization was observed, but no K localization

We have scanned over the surfaces to visualize the morphology depended Na^+^ distribution by using ESEM. Figure 4 showed Na and K contour maps over the surfaces after 7 and 9 days exposure to100 mM NaHCO_3_. Compartmented Na^+^ was found underneath the epicuticular surfaces, but no K^+^. The surface morphologies over the high Na^+^ accumulations were different from those over the control surfaces. It seemed that the degree of Na^+^ was associated with the morphological changes of the epicuticular surface.

### Absorption comparison of cytosolic Na^+^ and K^+^ in rice and *p. tenuiflora*

**Fig. 5:**
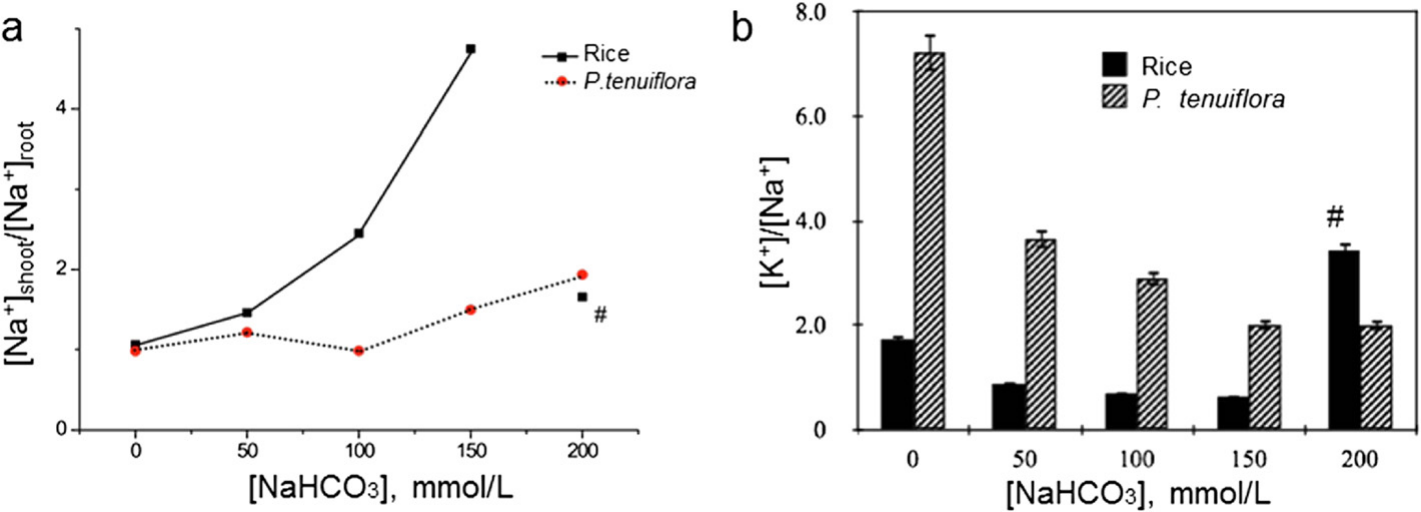
Atomic absorption spectroscopy under different NaHCO_3_ stress; (**a**) the ratio distribution of the absorbed Na^+^ ions from root to shoot, [Na^+^]_shoot_/[Na^+^]_root_ of rice and *P. tenuiflora*. **b** [K^+^]/[Na^+^] ratio distribution of rice and *P. tenuiflora* shoot. (#; unusual data by Na^+^ toxicity in shoot)

Plant usually balances at low cytosolic [Na^+^], and a cytosolic [K^+^]/[Na^+^] >1 [12]. Figure 5 showed that Na^+^ influx from the high external [NaHCO_3_] altered the [K^+^]/[Na^+^] in the rice. Na^+^ distribution ratio of shoot to root for rice also increased significantly from 0 mM to 150 mM NaHCO_3_ stress, appearing as [Na^+^]_shoot_/[Na^+^]_root_ > 1 (Fig. 5a). It seems the absorbed Na^+^ ions from root were transported to the shoot. Consequently, the [K^+^]/[Na^+^] ratios in rice shoots decreased gradually lower than 1 (Fig. 5b). Transported Na^+^ ions were accumulated to be toxic effects in rice shoot. At the 200 mM NaHCO_3_ stress, [Na^+^]_shoot_ dropped dramatically (# marked in Fig. 5a and b). This [Na^+^] decrement may be caused from a dye-functioned rice (yellowish colored shoot) due to high toxicity. Localized NaCl swollen corn shapes and columns formed by rupturing and/or leaking highly accumulated NaCl were correlated to decreased cytosolic [Na^+^] at extremely high NaHCO_3_ stress.

For *P. tenuiflora*, Na^+^ concentrations of both root ([Na^+^]_root_) and shoot ([Na^+^]_shoot_) were always balanced well at a very low level. This stable [Na^+^]_root_/[Na^+^]_shoot_ indicated that the external saline stress barely affected *P. tenuiflora*, which was different from those for rice. Figure 5b showed that ratio of [K^+^]/[Na^+^] decreased for both *P. tenuiflora* and rice, but for *P. tenuiflora*, [K^+^] was twice higher than [Na^+^] as NaHCO_3_ stress increased, while [K^+^] became almost 5 times lower than [Na^+^] for rice.

### Surface morphology and EDX profiles for *P. tenuiflora*

**Fig. 6:**
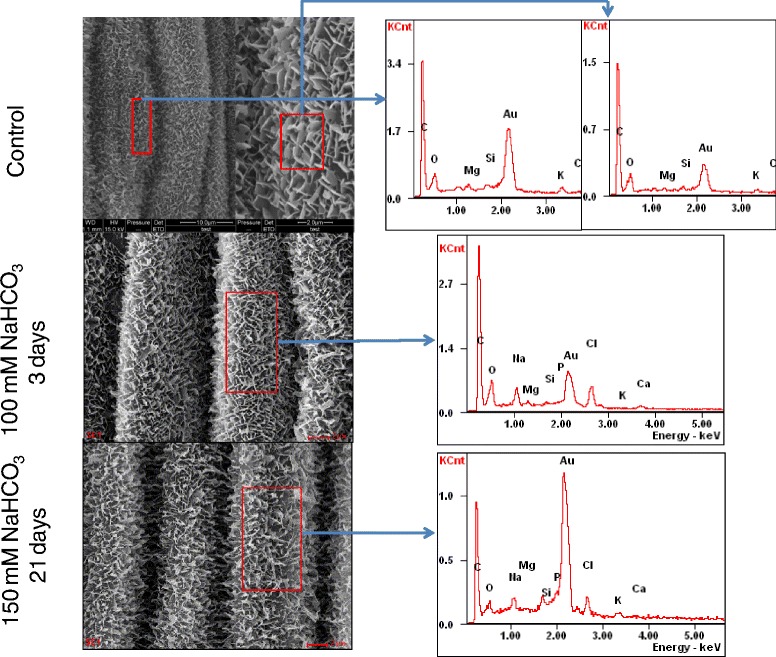
ESEM images of *p. tenuiflora* leaf surface experienced with 0 mM, 100 mM NaHCO_3_ for 3 days, and 150 mM NaHCO_3_ for 21 days. EDX spectra over the different spots (marked box on ESEM images) showed no significant changes

Figure 6 showed the epicuticular morphologies of *P. tenuiflora* with EXD characterization. Interestingly, epicuticular surface morphology of *P. tenuiflora* had no remarkable changes with experiencing the NaHCO_3_ stress (Fig. 6). Morphology of waxy crystal network on the *P. tenuiflora* epidermis surfaces was similar as that on the controlled rice leave surfaces. Even at high NaHCO_3_ stress, 150 mM for 21 days exposure, the wax and surface morphologies had no remarkable changes, EDX profiles also showed no remarkable changes of the element concentrations either, including Na^+^ and K^+^.

## Discussion

The waxy layer on the leaf surfaces prevents both molecular uptake and efflux. For both rice and *P. tenuiflora,* crystalline wax distributed randomly to form the micro-networks over the epicuticular surfaces. There was no distinction in the structure and density of wax crystal networks between different types of epicuticular cells, but no wax crystal networked coverage on the TC surfaces (Fig. 1a).

As both rice and *P. tenuiflora* exposed to NaHCO_3_ stress, no noticeable changes of the crystalline wax networks were observed. It seemed that the external NaHCO_3_ stress did not alter the wax synthesis metabolisms. However, surface morphologies of rice leaves had significant changes as Na^+^ localization increased. The surface deformation might be caused mainly by the Na^+^ accumulation under NaHCO_3_ stress. Surface deformations appeared as protruded surface bands and MSC, C and S-C for rice. The observed C and S-C on the disrupted-waxy surfaces of rice epidermis were composed mainly by NaCl. In addition, the excessive Na^+^ could be toxic to plant metabolism affecting its development and growth.

Under the NaHCO_3_ stress, atomic absorption spectra showed no noticeable increment of [Na^+^]_root_ for both rice and *P. tenuiflora.* It seemed that root had the capability to maintain the cytosolic hemostasis at low [Na^+^]_root_/[K^+^]_root_. However, for rice, [Na^+^]_shoot_ increased as the external salt stress increased in terms of exposure time and concentration of NaHCO_3_. The absorbed Na^+^ ions from root seemed to be transported to shoot and to be accumulated in the tissue cells of shoot. Excessive Na^+^ in the rice shoot caused the increment of [Na^+^]_shoot_/[K^+^]_shoot_ which was toxic to its growth. Consequently, dysfunction of rice under the NaHCO_3_ stress began not from root but from the shoot. In general, high [Na^+^]_shoot_ in halophytes imply compartmentation into the vacuole to maintain the ion homeostasis. Our results showed the compartmented Na^+^ over epicuticular surfaces of NaHCO_3_ experienced rice, but no K^+^compartmentation (Fig. 4). However it was not clear whether the compartmentations were in the vacuole.

EDX element analysis showed no homogeneous [Na^+^] enhancement over the epicuticular surface of rice leave as NaHCO_3_ stress increased. Initially wart-like protuberance silica cells were enlarged and merged to the big protuberance silica cell (MSC) (Fig. 2b). These manners of morphological changes are very similar to those under the silicon treatments due to the accumulation of silicon [13, 14]. Silicon is predominantly deposited in wart-like protuberance silica cells of the epidermis. Our X-ray microanalysis spectra showed that Na presented highly on the enlarged/merged silica cells (Fig. 3c) with low Na X-ray counts around stomatal guard cell areas. It seemed that excessive Na accumulated in silica cells as a similar manner of Si accumulation.

Further enhanced NaHCO_3_ stress induced the swollen corn-shaped (S-C) dumps and/or columns (C) on the epicuticular surface (Fig. 2c). EDX element spectra showed that those localized morphologies contained mostly NaCl. The cubic crystals appeared on the cross-section surface were confirmed to be NaCl crystal (arrow mark in Fig. 2d). Interestingly, the surface of swollen (S-C) NaCl localization was covered with the wax crystals networks, but that of NaCl column (C) was not. It suggested that the swollen localizations were formed slowly without disrupting the wax crystalline networks. The holes of the surface of this swollen localization indicated the remaining silica cells without swelling. However, the NaCl columns were formed by NaCl leakage from the silica cells. Highly accumulated NaCl was also observed underneath of the cell wall where the swollen localizations were found (Figs. 2d and 3f). The localization or secretion of highly NaCl accumulation on the epicuticular surfaces may be corresponding to the sudden recovery [Na^+^]_shoot_/[K^+^]_shoot_ at 200 mM NaHCO_3_ as shown in Fig. 5.

Na accumulation dominated in the enlarged silica cells, while in the swollen and column, Na and Cl elements dominated. There were noticeable difference in the element composition, Na > Cl for the leaked NaCl columns and Na < Cl for the swollen NaCl. This difference may be correlated to the different manner of NaCl secretion. The portion of free Na^+^ was accumulated in intra-cuticle cell wall, and the NaCl crystals were excreted in the form of column and swollen dump. It seemed that the NaCl secretion occurred after dysfunction. It would be a great challenge to trigger the NaCl secretion before dysfunction to enhance the salt tolerance on rice leaves.

The results obtained from atomic absorption spectroscopy showed that highly concentrated Na^+^ ions on the rice leave might be transported from root to shoot. For rice, the transported Na^+^ ions were accumulated in shoot appearing as the increment of [Na^+^]_shoot_, [Na^+^]_shoot_/[Na^+^]_root_ > 1. For *P. tenuiflora*, we did not observe the secreted Na^+^ ions on the surface of its leaf, but there might be some mechanisms to maintain the ionic homeostasis, [Na^+^]_shoot_/[K^+^]_shoot_ < 1.

## Conclusions

With the increase in NaHCO_3_ stress concentrate and time, there were no significant changes on the morphology of the waxy crystal networks for both rice and *P. tenuiflora* epidermis, however the epicuticular morphology of rice leave altered dramatically. MSC, S-C and C appeared as NaHCO_3_ stress increased. These new morphologies were correlated with the Na^+^ and Cl^−^ accumulations.

## Methods

### Plant cultivation and treatment

*P. tenuiflora* and *O. sativa L*. cv. (Nipponbare rice) were cultivated in hydroponics with a temperature of 25 ~ 28 °C, light exposure of 6000 lx, optical cycle of 16/8 h (day/night), and relative humidity of 60 %. Water was changed every 5 days. During the process of cultivation, 1/4 Hoagland was used for 2 to 3 days and for the rest of the time distilled water was used.

Ninety *P. tenuiflora* and rice seedlings of trefoil stage were selected and divided randomly into three different groups, 30 for each. The roots were washed with distilled water. The seedlings were then placed under 0 mM, 50 mM, 100 mM, 150 mM and 200 mM NaHCO_3_ stress for 1, 3, 5, 7, 9 and 21 days for *P. tenuiflora*, and under 0 mM, 50 mM, 100 mM, 150 mM and 200 mM NaHCO_3_ stress for 1, 3, 5, 7, and 9 days for rice, respectively.

### ESEM and EDX observations

The middle sections of the second true leaves of *P. tenuiflora* and rice seedlings were taken randomly in the treatment group and the control group. They were cut into 3 ~ 5 mm segments, quickly fixed in 3 % glutaricdialdehyde. The dehydrated samples with a vacuum dryer were coated with grain-size gold particles by using sputter coater (SCD005, Bal-Tec GmbH, Germany). The epicuticular surfaces of rice leaves were then visualized with an environmental scanning electron microscopy (ESEM, Quanta-200, Fei Co., USA). Wax composition and epicuticular chemical composition were recorded by EDX during ESEM imaging. X-rays were collected with a detector at the takeoff angle of 30°.

### [Na^+^] and [K^+^] measurements

Saline stress was applied under 50 mM, 100 mM, 150 mM and 200 mM NaHCO_3_. Sample groups were cultivated for 5 days, and the sample materials were removed from the stress solution, washed two times with distilled water to remove surface salt ions. The prepared shoot and root were placed on dry filter paper for absorbing moisture and dried in an oven at 105 °C for 10 min. The dried samples were grounded and digested in the 10 ml nitric acid and 1 ml perchloric acid solution. Using 220FS atomic absorption spectrophotometer (Varian, USA), [Na^+^] of root and shoot were measured.
